# Case Report: Juvenile dermatomyositis complicated by acute myeloid leukemia: achievement of sustained remission through hematopoietic stem cell transplantation

**DOI:** 10.3389/fimmu.2026.1776236

**Published:** 2026-04-23

**Authors:** Pan Fu, Kai Chen, Qing Liu, Bing Zou, Zhen Wang, Dan Wang, Hui Jiang, Na Zhang, Jingbo Shao

**Affiliations:** Department of Hematology and Oncology, Shanghai Children’s Hospital, School of Medicine, Shanghai Jiao Tong University, Shanghai, China

**Keywords:** acute myeloid leukemia, children, hematopoietic stem cell transplantation, juvenile dermatomyositis, sustained remission

## Abstract

Juvenile dermatomyositis (JDM), a pediatric subtype of idiopathic inflammatory myopathy, is a relatively rare rheumatic and immune system disease among children. Although paraneoplastic dermatomyositis (DM) has been documented in adult malignancies, its association with acute myeloid leukemia (AML) in pediatric populations remains exceptionally rare. We present a male patient diagnosed with JDM at age 6, characterized by Gottron’s papules and muscle weakness. Despite receiving sequential immunosuppressive therapies and biological agents, the disease followed a relapsing-remitting course over 9 years. At age 15, he developed persistent knee arthralgia accompanied by intermittent fever lasting 8 days. Hematologic evaluation revealed thrombocytopenia (platelet count: 76 ×10^9^/L) and leukocytosis (white blood cell count: 36.46 ×10^9^/L), prompting bone marrow aspiration confirmed AML diagnosis (French-American-British M2 subtype). Following induction chemotherapy and two consolidation cycles, allogeneic hematopoietic stem cell transplantation (HSCT) from a matched unrelated donor was successfully performed. Notably, both JDM symptoms and AML achieved sustained remission at 1-year follow-up post-HSCT. This case suggests that HSCT may represent a potential therapeutic approach for refractory autoimmune-hematologic malignancy overlap syndromes, though further studies are required to validate its efficacy.

## 1 Introduction

Juvenile dermatomyositis (JDM), a rare pediatric autoimmune disorder, has been reported occasionally in association with hematological malignancies. Here, we present the first documented case of JDM progression to acute myeloid leukemia (AML) with sustained remission following hematopoietic stem cell transplantation (HSCT).

In August 2014, a six-year-old Chinese boy was admitted to an external hospital with a three-year history of facial rash and a one-month history of weakness in both lower limbs. Laboratory tests revealed elevated levels of muscle enzymes and myoglobin. Electromyography indicated myopathic changes, and a biopsy of the right gastrocnemius muscle showed partial degeneration of striated muscle fibers, loss of striation, and interstitial fibrosis. Myositis-associated autoantibodies (MAA) were not tested. A diagnosis of juvenile dermatomyositis was made. The patient was subsequently treated with prednisone and methotrexate. However, after relapse of the skin rash, the regimen was changed to mycophenolate mofetil (MMF) and methylprednisolone in May 2015. Two years after diagnosis (October 2016), he developed progressive subcutaneous calcification and skin ulceration, leading to adjustment of the treatment to include hydroxychloroquine and intravenous immunoglobulin (IVIG) together with the methotrexate and prednisone. Despite these interventions, the cutaneous lesions continued to recur. In August 2017, he underwent high-dose glucocorticoid pulse therapy, high-dose IVIG, methotrexate (MTX), cyclosporine, thalidomide, and alendronate. Cyclosporine was discontinued in November 2018 due to hypertension and mild cataract formation, and was replaced by tacrolimus. By July 2020, the skin rash had worsened, accompanied by muscle pain in the upper arms and a significant rise in serum creatine kinase (CK) levels, leading to the addition of tofacitinib to the methylprednisolone and MTX regimen. In January 2021, the patient complained of sore muscles in his upper arms. The medication regimen was subsequently adjusted to oral hydroxychloroquine, prednisone acetate, thalidomide, and MTX, which the patient has been taking continuously since then.

**Figure 1:**
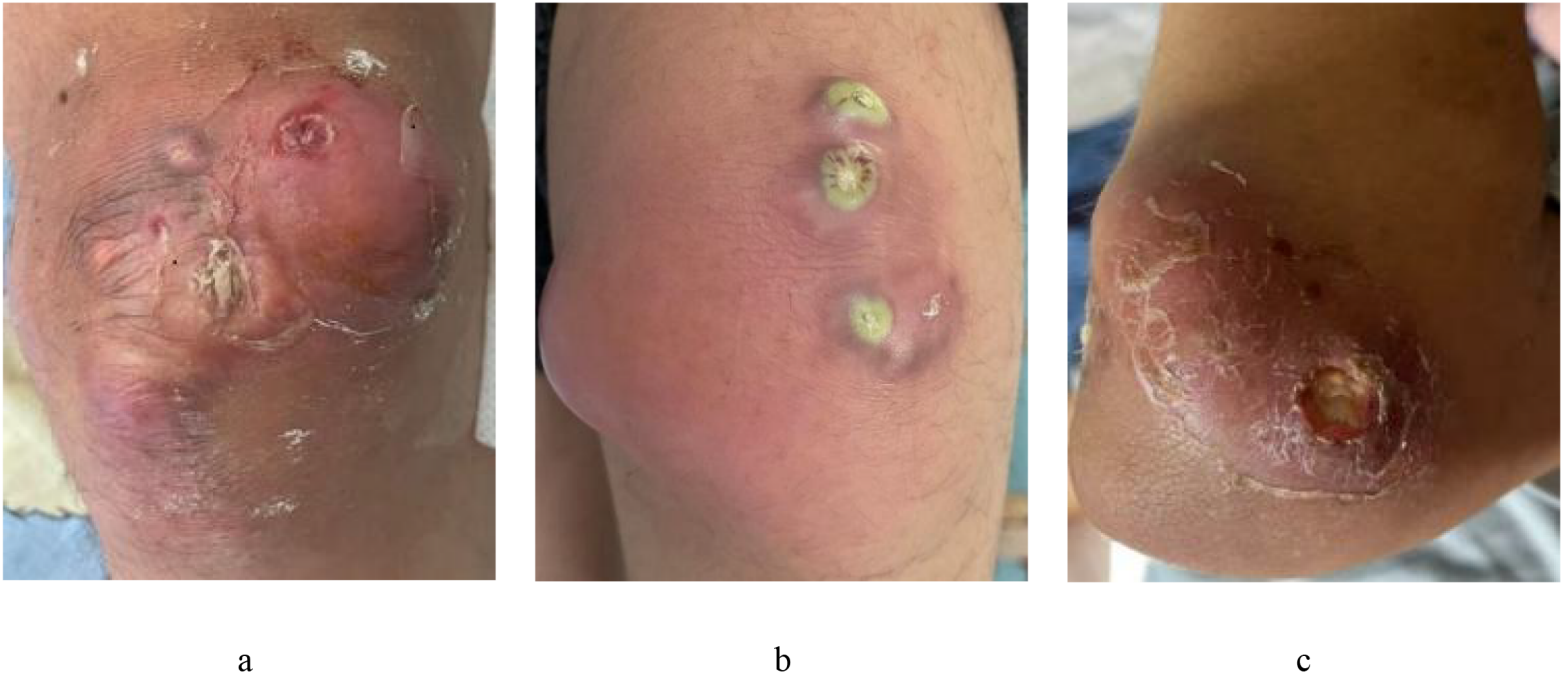
Skin lesions on the right knee joint **(a)**, left knee joint **(b)**, and left elbow joint **(c)** at 15 years of age.

In March 2024, the child was hospitalized due to knee-joint pain accompanied by intermittent fever that had persisted for eight days with no improvement following anti-infection therapy. On admission, his temperature was 36 °C, with a pulse of 85 beats per minute, respiration rate of 18 breaths per minute, and blood pressure of 119/75 mmHg. Physical examination indicated no superficial lymphadenopathy. Erythema, swelling, and ulceration were noted over the bilateral knee joints and the left elbow joint, along with cutaneous calcifications (**Figure 1**). A milky-white exudate mixed with yellowish discharge was observed from the lesions during joint flexion or extension. Cardiopulmonary auscultation revealed clear lung fields without rales and regular heart sounds of normal intensity. Abdominal palpation indicated hepatomegaly (+2 cm from the costal margin) with no splenomegaly. The results of the neurological examination were unremarkable, with the presence of normal muscle strength and tone in all extremities. He had an initial Childhood Myositis Assessment Scale (CMAS) of 42/52. Laboratory test findings indicated significant leukocytosis, with a white blood cell count of 36.46×10^9^/L, and thrombocytopenia, with a platelet count of 76×10^9^/L, while hemoglobin levels remained within the normal range at 123 g/L. A peripheral blood smear revealed 14% blasts. The levels of inflammatory markers were significantly elevated, with a C-reactive protein (CRP) level of 53 mg/L and an erythrocyte sedimentation rate (ESR) of 108 mm/h. Hyperferritinemia (ferritin, 3240.0 ng/mL) and elevated lactate dehydrogenase (LDH) of 1028 U/L (3.5×upper limit of normal) were observed. The levels of procalcitonin (PCT, 0.24 ng/mL) were mildly elevated, while those of creatine kinase (CK,<20 U/L) and myoglobin (<21.00 ng/mL) remained below the lower limit of detection. And there was no evidence of antibodies to extractable nuclear antigen (ENA) or antinuclear antibody (ANA). Bone marrow aspiration showed 7.5% abnormal promyelocytes, while immunophenotyping further revealed approximately 5.4% abnormal myeloblasts. Molecular analysis using fluorescence *in situ* hybridization (FISH) confirmed the presence of the *ETO/AML1* fusion gene associated with the t(8;21)(q22;q22) translocation in 92.3% of cells. French-American-British classification M2 AML type was diagnosed.

**Figure 2:**
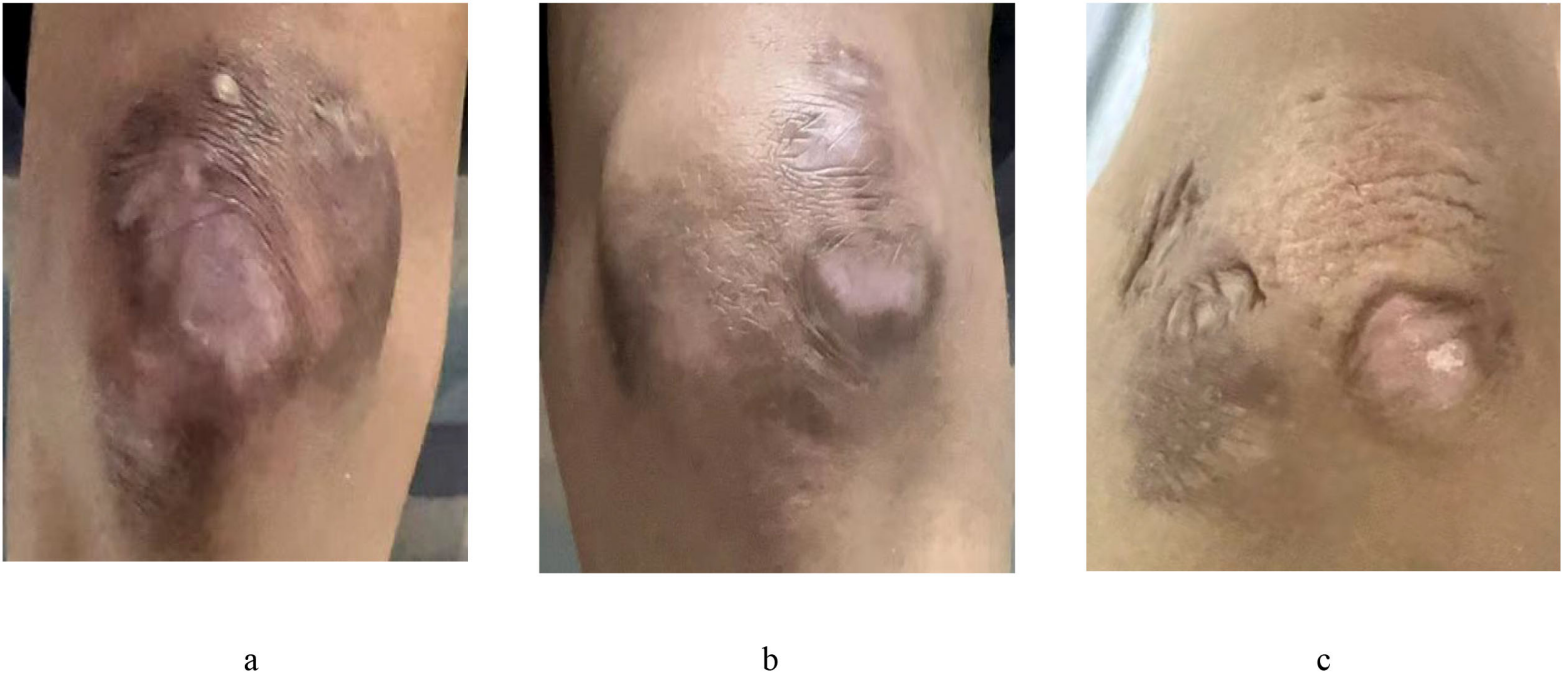
Skin lesions on the right knee joint **(a)**, left knee joint **(b)**, and left elbow joint **(c)** at 17 years of age.

The management of the case posed significant challenges due to the combination of subcutaneous calcification with ulcerative lesions and the superimposition of infection. Due to the risk of infection exacerbation resulting from chemotherapy-induced myelosuppression, an antimicrobial-intensive regimen was initiated alongside a chemotherapy protocol consisting of daunorubicin, cytarabine (Ara-C), and homoharringtonine (HHT). Bone marrow evaluation on day 28 post-chemotherapy confirmed complete remission ((measurable residual disease [MRD] <0.1%), with a decrease in the *AML1-ETO* transcript levels from 190.21% at diagnosis to 1.18%. A peripherally inserted central catheter (PICC) was placed in the left arm on day 2 of chemotherapy. Swelling of the left arm was noted two days after catheter insertion, with ultrasonography confirming catheter-related thrombosis, necessitating removal of the PICC. Subsequent placement of the PICC in the right arm was complicated by the development of ipsilateral swelling and thrombosis within 48 hours. Treatment with intravenous dexamethasone and oral edoxaban was effective in resolving these symptoms, and radiological resolution of the thrombus was confirmed on follow-up ultrasonography. The second treatment cycle (mitoxantrone + Ara-C+ HHT) reduced *AML1-ETO* transcript levels to 0.32%, with achievement of molecular negativity after the third treatment cycle (cladribine+Ara-C+G-CSF+venetoclax). Unrelated donor allogeneic hematopoietic stem cell transplantation was performed on August 28, 2024. The conditioning regimen followed our institution’s protocol for relapsed/refractory cases and was based primarily on FLAG followed by busulfan (Bu) and melphalan (Mel). Prophylactic treatment for graft versus host disease consisted of ATG, cyclosporine (CsA), mycophenolate mofetil (MMF), and a short course of MTX. The patient received 9.1×10^8^/kg of nucleated cells and 11.1×10^6^/kg of CD34+ cells isolated from the peripheral blood of the donor. Engraftment was observed, indicated by a complete blood cell count with an absolute neutrophil count of > 1.0×10^9^/L on day +11 and platelet implantation on day +17. GVHD prophylaxis was administered with cyclosporine following transplantation. This treatment was tapered off from two months after transplantation, with gradual discontinuation at 13 months due to intermittent abdominal pain that had begun at four months post-transplant. At nine months post-transplant, recurrent gastrointestinal symptoms consistent with chronic intestinal GVHD led to the addition of belumosudil, resulting in clear resolution of the gastrointestinal symptoms. The patient is currently receiving tapered doses of oral belumosudil. At 18 months after transplantation, the patient had maintained complete continuous remission with MRD negativity and no detection of *ETO/AML* fusion gene transcripts by qRT-PCR. There was no recurrence of dermatomyositis (DM) manifestations; specifically, the patient exhibited no muscle weakness and no new cutaneous lesions or calcification, with only the remains of earlier cutaneous changes observed over the joints of the extremities (**Figure 2**). Serum levels of creatine kinase (CK), aspartate aminotransferase (AST), and lactate dehydrogenase (LDH) were all within normal reference ranges.The CMAS score was 52, and the PedsQL 4.0 Generic Core Scales showed a total score of 79.

## Discussion

Dermatomyositis is a rare autoimmune inflammatory condition affecting the skin and muscles. The disease occurs in both adults and children (1), affecting approximately 9.63 per million adults and 1.9–4.1 per million children (2). The mean age of disease onset in children was found in one study to be 87.6 months (3), while another report described a mean age of onset of 81.97 months (4). The risk of calcinosis and ulceration is higher in children. Numerous studies have consistently demonstrated a higher prevalence of malignant neoplasms among individuals diagnosed with polymyositis (PM) or DM (5).

There are few reports on AML associated with JDM. A systematic review identified 10 documented cases of DM with AML reported in the medical literature to date (6–15) (**Table 1**). In these 10 published cases, AML was reported to develop either concurrently with or after DM/PM onset. These 10 cases included two pediatric patients. Among the 10 cases, males were more frequently affected than females (7 males vs. 3 females). Notably, only four cases were reported to have achieved sustained remission, with the remaining patients exhibiting treatment failure (not in remission, relapse after remission, or death from complications). Five of the 10 patients (50%) had prior exposure to cytotoxic agents, including azathioprine, cyclosporine, MTX, and chemotherapeutic drugs. In the present case, the boy developed AML 9 years after the initial diagnosis of JDM. Notably, he was given prolonged administration of immunosuppressive agents and cytotoxic drugs following the JDM diagnosis, which could lead to treatment-related carcinogenesis. However, there is no definitive evidence of a causal link between these therapies and AML development. As an alternative hypothesis, JDM may represent a paraneoplastic manifestation preceding AML; however, the temporal dissociation (9-year interval) between the autoimmune and malignant presentations challenges this interpretation. Therefore, we consider that there is a causal link between these therapies and AML development. Following three cycles of chemotherapy and HSCT, the patient achieved complete sustained remission of both JDM and AML, with no evidence of disease recurrence at the last follow-up.

**Table 1:** Case reports of AML associated with PM/DM.

Reference	Year	Sex	Age(years)	Latency of onset	AML subtype	History of cytotoxic drugs	Treatment before AML occurrence	Treatment for AML	Ending reported in the article
Evans and Hilton (6)	1964	F	30	0	M5	No	Steroids	6-mercaptopurine and prednisone	Maintained reasonably well
Goldstein et al. (7)	1978	M	34	11 months	AML(unclassified)	No	Steroids	Chemotherapy(daunomycin and cytosine arabinoside)	Died of pancytopenia, congestive heart failure, and sepsis caused by *Streptococcus pneumoniae*
Sugawara et al. (8)	1992	M	30	4 years	M3	Yes	Prednisolone was administered for DM. The patient was later diagnosed with testicular seminoma with pulmonary metastases, for which he received orchidectomy plus 4 courses of chemotherapy (cisplatin, etoposide, peplomycin, and prednisolone)	Chemotherapy(behenoyl cytosine arabinoside, daunorubicin, 6-mercaptopurine, and prednisolon)	Achieved CR 1 month after chemotherapy and remained in complete remission as of June 1991
Krishnan et al. (9)	1994	M	52	9 years	M0	Yes	Initial treatment with prednisone, which was replaced with azathioprine due to steroid-induced diabetic coma	Standard induction regime with daunorubicin and cytosine arabinoside, and two cycles of consolidation chemotherapy with daunorubicin and cytosine arabinoside	Relapsed after CR for two months
Ambrosone et al. (10)	1995	M	62	3 months	M2	No	Prednisone	Only cytosine arabinoside	Died from acute pulmonary edema
Arnold et al. (11)	1999	F	66	2 years	M2	Yes	Azathioprine and prednisolone	DAT chemotherapy (daunorubicin, cytosine arabinoside and 6-thioguanine); two courses of FLAG regimen (fludarabine, cytosine arabinoside, granulocyte-colony-stimulating factor)	Remission was not achieved with DAT chemotherapy. The FLAG regimen succeeded in inducing morphological and cytogenetic remission, but the patient relapsed after the second FLAG course and eventually died due to leukemic relapse
Carrera et al. (12)	2004	M	56	8 years	AML (unclassified)	Yes	Initial prednisone treatment failed; azathioprine + chloroquine were then added, resulting in no improvement and consequent replacement with prednisone + cyclosporine. High-dose IVIG was attempted for refractory DM, followed by cyclophosphamide + prednisone due to lack of IVIG efficacy	Induction chemotherapy with idarubicine, cytarabine, and etoposide	Achieved CR for both DM and AML and maintained remission for 1 year. A subsequent concurrent relapse of DM and AML was observed, and he died of pulmonary bleeding at 66 years old.
Shen JK et al. (13)	2008	M	30	12 months	M3	No	Prednisone	ATRA	Remained stable
Shrivastav A et al. (14)	2010	M	14	0	M2	No	Prednisolone	Induction therapy with daunorubicin and cytarabine	Died of sepsis on third day of chemotherapy.
Cannon L, et al. (15)	2020	F	6	18 months	M3	Yes	Methylprednisolone + IVIG after diagnosis; then oral steroids + MTX + hydroxychloroquine	Chemotherapy	Remained in remission 8 months post-therapy

F, Female; M, Male; CR, complete response.

In summary, allogeneic hematopoietic stem cell transplantation may represent a potential therapeutic strategy for patients with dermatomyositis and leukemia.

## Data Availability

The datasets presented in this article are not readily available because of ethical and privacy restrictions. Requests to access the datasets should be directed to the corresponding authors.
